# Clinical, therapeutic and prognostic differences between male and female patients with breast cancer—a comparison of 2510 men and 307,634 women in a registry-based study in Germany

**DOI:** 10.1007/s00432-025-06220-y

**Published:** 2025-06-03

**Authors:** Marion Graf, Michael Gerken, Monika Klinkhammer-Schalke, Simone Schrodi, Armin Pauer, Karla Geiss, Olaf Ortmann, Elisabeth C. Sturm-Inwald

**Affiliations:** 1 Tumor Center Regensburg—Center for Quality Management and Health Services Research, University of Regensburg, Regensburg, Germany; 2 Bavarian Cancer Registry, Regional Centre Regensburg, Bavarian Health and Food Safety Authority, Regensburg, Germany; 3 Bavarian Cancer Registry, Bavarian Health and Food Safety Authority, Munich, Germany; 4 Bavarian Cancer Registry, Bavarian Health and Food Safety Authority, Erlangen, Germany; 5 Department of Gynecology and Obstetrics, University Medical Center Regensburg, Landshuter Straße 65, 93053 Regensburg, Germany

**Keywords:** Male breast cancer, Therapy, Prognosis, Prognostic factors, Overall survival, Cancer registry

## Abstract

**Purpose:**

The aim of the present study was to compare patient, tumor characteristics and prognostic factors as well as diagnostics and therapies between men and women with breast cancer. The rates of primary distant metastases, contralateral second tumors, overall survival (OS), recurrence, and recurrence-free survival (RFS) were analyzed and compared between men and women.

**Methods:**

This retrospective cohort study included patient data from 18 clinical cancer registries in Germany (2000–2018). Differences in risk factors and short-term endpoints were analyzed via univariable and multivariable binary logistic regression analyses. OS, RFS, and the rate of subsequent second tumors were examined via Kaplan‒Meier, univariable and multivariable Cox regression methods.

**Results:**

Compared with women, male patients with breast cancer presented a significantly greater risk of unfavorable prognostic factors, such as advanced stage, lymphatic invasion, and more primary distant metastases. While sentinel lymph node biopsy and HER-2 testing were comparable, treatment rates for men were 9.5–29.0% lower than those for women. In multivariable analyses, men had a 1.32-fold increased risk of death (95% CI 1.24–1.41; *p* < 0.001). The risk of recurrence/mortality was significantly increased by a factor of 1.531 (95% CI 1.43–1.65; *p* < 0.001). Adjustment for therapy in a multivariable regression model did not significantly affect the risk of death. Nevertheless, men had a survival benefit from systemic therapies comparable to that of women.

**Conclusion:**

Neither patient and tumor characteristics nor differences in therapy could completely explain the difference in mortality between men and women. Differences in lifestyle or biological factors could play a role.

**Supplementary Information:**

The online version contains supplementary material available at 10.1007/s00432-025-06220-y.

## Introduction

Breast cancer is the most common cancer in Germany and worldwide. In Germany, approximately 70,000 people are diagnosed with breast cancer every year. Men have a lifetime risk of developing breast cancer of 0.1%, whereas the lifetime risk for women is 12.4% (Ronckers et al. 2023). The proportion of men among all new breast cancer cases is approximately 0.6–1.0% (Ronckers et al. 2023; Miao et al. 2011; Giordano et al. 2004; Anderson et al. 2010; Schoffer et al. 2024). Owing to the rarity of this disease, there is no screening for the early detection of breast cancer in men. Therefore, men are usually diagnosed after the onset of symptoms (Harlan et al. 2010; Aid and AWMF 2021). Several studies have reported that men are often diagnosed at advanced stages (Giordano et al. 2004; Elimimian et al. 2021; Gnerlich et al. 2011; Aggarwal et al. 2021; Joshi et al. 1996; Liu et al. 2018; Wang et al. 2019). Other reasons for delayed diagnosis include a late consultation due to a lack of knowledge about male breast cancer in the population (Aid and AWMF 2021). To date, most of the knowledge is based on retrospective analyses and small numbers of cases. Hence, treatment recommendations for men with breast cancer have been derived mainly from those for postmenopausal women (Aid and AWMF 2021; Korde et al. 2010; Ruddy and Winer 2013). There are conflicting results in the literature regarding the prognosis of men with breast cancer. The Robert Koch Institute (RKI) reported a higher survival rate for women than for men with breast cancer in Germany, with an absolute 5-year survival rate of approximately 80% for women and approximately 60% for men (Ronckers et al. 2023). Some studies have suggested that men and women have similar prognoses in terms of breast cancer-specific survival or survival at the respective stages (Giordano et al. 2004; Cutuli et al. 1995). In contrast, a study by Wang et al. revealed that men had a greater risk of death than women with breast cancer at any stage, even after adjusting for clinical and therapeutic factors, age, ethnicity, and access to medical care (Wang et al. 2019). The aim of the present study was to compare patient and tumor characteristics and prognostic factors between men and women with breast cancer. Additionally, a comparison of diagnostics and therapies was carried out in the respective indication groups for men and women. The rates of primary distant metastases, contralateral second tumors, overall survival (OS), recurrence, and recurrence-free survival (RFS) were analyzed and compared between men and women. Any differences in prognosis between men and women were further analyzed to investigate differences in clinical factors and treatment and to determine whether sex was an independent risk factor.

## Materials and methods

### Study population

In this retrospective cohort study, patient data from the Association of German Tumor Centers (Arbeitsgemeinschaft Deutscher Tumorzentren—ADT) from 18 clinical cancer registries in Germany (federal states Mecklenburg-Western Pomerania, Brandenburg, Saxony-Anhalt, Saxony, Thuringia, Hesse, Bavaria and Baden-Wuerttemberg) were analyzed between January 1 st, 2000, and December 31 st, 2018.

**Fig. 1:**
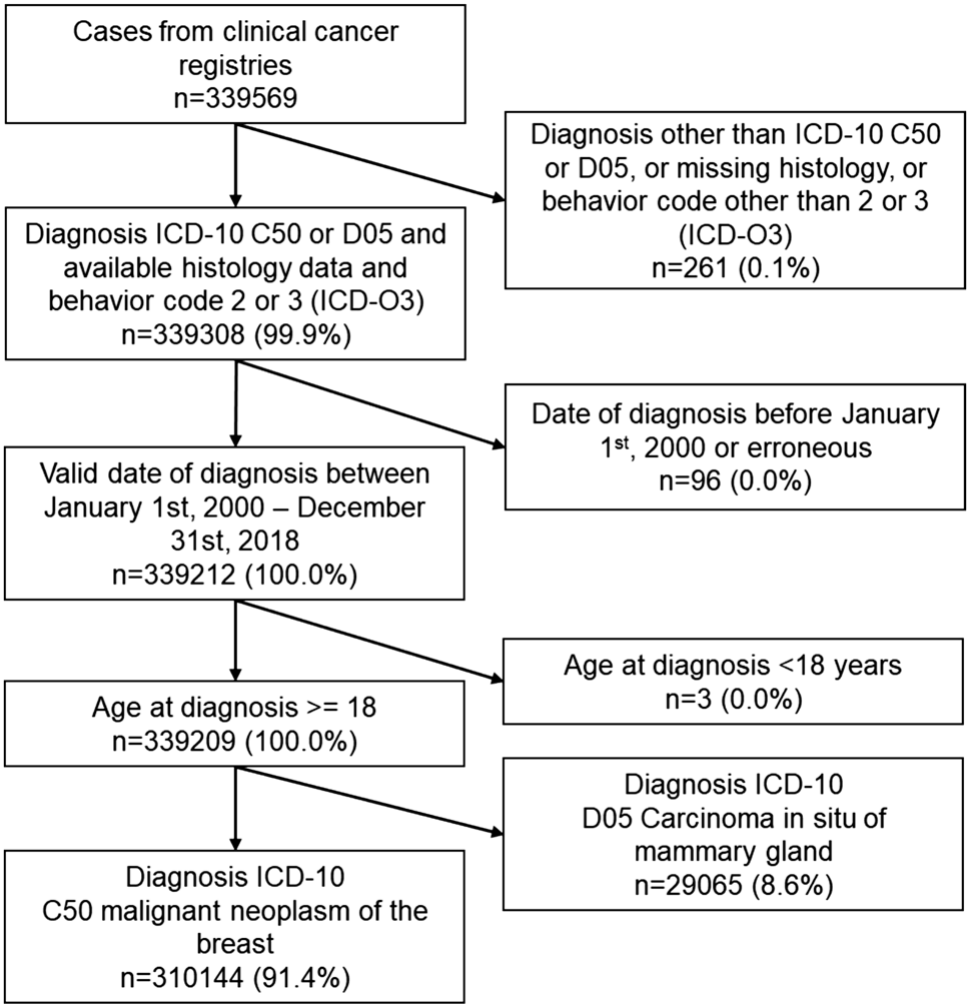
Inclusion and exclusion criteria

Figure 1 shows the inclusion and exclusion criteria. Patients without International Statistical Classification of Diseases and Related Health Problems (ICD) ICD-10 C50 or D05 or missing histology or behavior codes other than 2 or 3 in the morphology code of ICD-O3 were excluded (n = 261). Three patients aged < 18 years were also excluded. A total of 8.6% (n = 29,065) of the remaining cases were classified as carcinoma in situ of the mammary gland and were excluded from the analyses. The final study population consisted of 310,144 male and female patients with malignant neoplasms of the breast (ICD-10 C50).

### Statistical analyses

Patient and tumor characteristics, period of diagnosis, age at diagnosis, side localization, histological classification, Union Internationale contre le Cancer (UICC) stage, grading, lymphatic and venous invasion, hormone receptor status, human epidermal growth factor receptor 2 (HER2) status, Ki-67, and molecular subtype were compared between men and women.

To test for differences in the mean age at diagnosis between men and women, Student’s t test was used, and in the case of a nonnormal distribution, the Mann‒Whitney U test was used. Pearson’s chi-square test and univariable and multivariable binary logistic regression were used to test whether the independent variables were distributed differently between men and women.

The median follow-up time, 5-year OS, cumulative recurrence rate, and 5-year RFS were estimated via the Kaplan‒Meier method. End-points were calculated from the date of diagnosis to the first event. The follow-up period and survival times were right censored, and December 31 st, 2018, was used as the cutoff date. A locoregional event was defined as any epithelial breast cancer or DCIS in ipsilateral breast tissue or ipsilateral axillary, infraclavicular, supraclavicular, internal mammary, parasternal, or intramammary lymph nodes after R0 resection by mastectomy or breast conserving therapy. In contrast, a second primary breast cancer signifies any epithelial breast cancer in the contralateral breast independently from lymph node metastases on that side. In the analyses of cumulative recurrence rates and rates of subsequent contralateral breast cancer censoring by date of death was taken into account. Differences in survival were tested for significance via the log-rank test (Mantel‒Cox). Survival, cumulative recurrence rates and RFS in the total cohort and in the subgroups, as well as the rates of subsequent second tumors, were examined via multivariable Cox regression with hazard ratios (HRs) and 95% confidence intervals (95% CIs) for the risk of men versus women, adjusting for age at diagnosis, side localization, histology, grading, lymphatic/venous invasion, hormone receptor status, HER2 status, Ki-67, and period of diagnosis. The influence of the therapies on sex-related differences in survival was analyzed by comparing the HRs of men and women with and without adjustment for therapy via multivariable Cox regression analyses. The influence of the therapies on survival was examined separately for men and women in a multivariable Cox regression with adjustment for patient and tumor characteristics. Binary logistic regression was used to analyse the OR of simultaneous second tumors. Recurrence rates and RFS were evaluated only in stage I–III and R0-resected patients. The rate of contralateral second tumors between men and women was not considered in tumors but rather in patients. Second tumors diagnosed less than 90 days after the first tumor were classified as simultaneous second tumors, and subsequent tumors were classified otherwise.

In order to adjust for the lower life expectancy of men compared to women, relative survival (RS) rates were estimated. Relative or net survival is a surrogate measure for cancer-specific survival and has the major advantage of not requiring information on cause of death, which is available in German cancer registry data, but presumably not sufficiently accurate. The cumulative relative survival rate is defined as the ratio of the observed overall survival rate in the patient group and the expected survival rate of a comparable group from the general population matched with respect to age at diagnosis and calendar year of diagnosis, in this context separately for men and women. For this comparison the official German life tables from 2000 to 2018 were used, stratified according to age, sex, and calendar year.

Furthermore, we used period survival analysis to provide more up-to-date measure of relative survival by using the most recent interval survival estimates of patients diagnosed in different calendar years (Brenner and Hakulinen 2002). The analysis was performed for consecutive cohorts by year of diagnosis to show the development of relative survival over years. Hereby, a 5-year period was applied, and trends were shown for the center year of the period, for instance 2012 shows the survival for the period 2010 to 2014.

The software SURVSOFT (Geiss et al. 2009) was applied for calculating overall survival choosing the standard life table (actuarial) method with one-year time intervals, for calculating relative survival choosing the method of Ederer (Ederer and Heise 1959) in order to estimate expected survival, and to estimate period survival (Brenner and Hakulinen 2002).

The level of significance was set at 0.05 for all the statistical tests. Calculations were performed with SPSS version 26 (IBM Corp., SPSS for Windows, Armonk, NY, USA).

## Results

### Patient and tumor characteristics

According to the inclusion criteria, 310,144 patients with malignant neoplasms of the breast were extracted from the total data pool (Fig. 1). A total of 0.8% (n = 2510) of these patients were male, and 99.2% (n = 307,634) were female. Supp. Table A shows the distributions of patient and tumor characteristics. The mean age at diagnosis for men (mean age 68.4 years, median 69.3 years, range 22.3–100.1 years) was significantly greater than that for women (mean age 63.0 years, median 63.5 years, range 18.2–107.5 years). Advanced stages were proportionally more common in men. The proportion of men with lymphatic invasion was significantly greater than that of women (32.3% vs. 22.1%). Positive hormone receptor status (78.3% vs. 69.6%) and luminal B tumors (17.3% vs. 9.8%) were more common in men than in women. The proportions of luminal A, HER2-enriched, and triple-negative tumors were lower in men.

In the multivariable binary logistic regression analysis, male patients with breast malignancies had a significantly greater risk of unfavorable prognostic factors, such as advanced stage and lymphatic invasion, than women did (Table 1). In addition, men showed a relative increase in the number of cases from 2012, a greater age at diagnosis, more often positive hormonal receptor status, negative HER2 status, and more often a Ki-67 ≥ 25%.

**Table 1:** Multivariable logistic binary regression with odds ratios for the risk men (n = 2,510) versus women (n = 307,634) with reference to patient and tumor characteristics

		p^a^	Odds Ratio (OR)	95% CI for OR	
				Lower limit	Upper limit
Period of diagnosis	2000–05	<.001	Ref.^b^		
	2006–11	.111	1.10	.98	1.25
	2012–18	<.001	1.59	1.39	1.82
Age at diagnosis (years)	0–49	<.001	Ref		
	50–59	<.001	1.63	1.36	1.95
	60–69	<.001	2.96	2.52	3.48
	70–79	<.001	3.53	3.01	4.14
	≥ 80	<.001	2.57	2.15	3.07
Side localization ICDO-3	Left	.159	Ref		
	Right	.023	.91	.84	.99
	Both sides	.997	.00	.00	
	n. s.^c^	.763	.93	.57	1.50
Stage UICC	I	<.001	Ref		
	0	.089	.45	.18	1.13
	II	<.001	1.60	1.44	1.79
	III	<.001	2.44	2.14	2.78
	IV	<.001	1.92	1.63	2.26
	X/n. s	<.001	2.03	1.71	2.40
Grading	G1	.026	Ref		
	G2	.054	1.14	1.00	1.30
	G3	.120	1.13	.97	1.32
	GX/n. s	.003	1.41	1.13	1.77
Lymphatic invasion	0	<.001	Ref		
	1	<.001	1.32	1.19	1.47
	X/n. s	.578	.95	.79	1.14
Venous invasion	0	.772	Ref		
	1	.627	1.05	.87	1.27
	X/n. s	.548	1.05	.89	1.25
Hormone receptor (HR) status	Negative	<.001	Ref		
	Positive	<.001	5.04	3.96	6.42
	n. s	<.001	4.26	3.28	5.53
HER2 status	Negative	<.001	Ref		
	Positive	.002	.80	.69	.92
	n. s	.007	1.20	1.05	1.37
Ki-67	Low risk (< 25%)	<.001	Ref		
	High risk (≥ 25%)	.001	1.26	1.09	1.45
	n. s	.001	1.23	1.09	1.39

^a^The p-values in the table given in the reference category refer to the entire variable

^b^Ref.: Reference

^c^N.s.: not specified

### Primary distant metastases

Men (m) had more primary distant metastases than women (w) (m: 10.4%, w: 7.3%). In both men and women, the most frequently reported sites of primary distant metastases were bone (m: 4.4%, w: 3.0%), lung (m: 2.9%, w: 1.4%), and liver (m: 1.4%, w: 1.3%) (Supp. Fig. A). Univariable and multivariable logistic regression revealed a significantly greater risk of primary distant metastases in men than in women (univariable: *p* < 0.001; multivariable: odds ratio (OR) = 1.17, 95% CI 1.02–1.34; *p* = 0,025). After multivariable adjustment for age at diagnosis, side localization, histology, grading, lymphatic/venous invasion, hormone receptor status, HER2 status, Ki-67, and period of diagnosis, the ORs for men versus women were significantly greater in terms of localization in the lungs (OR = 1.61, 95% CI 1.27–2.04; *p* < 0.001) and skin (OR = 1.76, 95% CI 1.07–2.90; *p* = 0.026).

### Sentinel lymph node biopsy and HER2 testing

The sentinel lymph node biopsy and HER2 testing in men and women were comparable. A total of 88.3% of men and 89.8% of women with clinically inconspicuous lymph nodes (cN0) underwent the indicated sentinel lymph node biopsy (*p* = 0.360, Supp. Table B). During the observation period, HER2 testing increased in both men and women (Supp. Fig. B). HER2 analysis was performed for 81.7% of men and 83.5% of women (*p* = 0.015, Supp. Table C).

### Treatment in stage I-III and R0 patients

Therapies were analyzed in patients with stage I-III disease and local R0 status. Breast-conserving therapy (BCT) was the most common surgery in women (72.1%). In contrast, only 13.9% of men underwent BCT. Mastectomy was the most common definitive surgery performed in 83.3% of men (Supp. Table D).

With respect to systemic therapies, the collective was restricted to patients with stage I–III disease with local R0 status after surgery. With respect to systemic therapies, the treatment rates for men were 9.5–29.0% lower than those for women. A total of 68.0% (n = 1050) of men and 75.2% (n = 130,140) of women with positive hormone receptor status received the indicated endocrine therapy. As antibody therapy with trastuzumab has been approved since 2006 for the adjuvant setting, analyses for antibody therapy were performed from 2006 onward. From 2006 to 2018, 45.9% (n = 61) of men and 60.2% (n = 13,244) of women with HER2-positive disease received the indicated antibody therapy. Among patients with an indication for CHT (negative hormone receptor status and/or positive lymph nodes and/or grade G3/4 and/or age < 35 years), 50.5% (n = 506) of men and 62.0% (n = 74,617) of women received CHT.

### Overall survival in stage I-IV patients

**Fig. 2:**
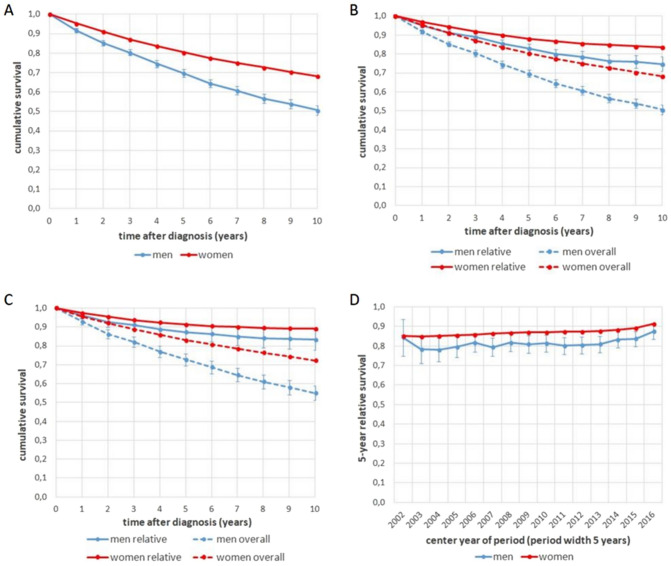
Comparison of overall, relative and period survival in men and women: **A** Cumulative probability of overall survival by sex, **B** comparison of relative and overall survival in men and women, **C** comparison of relative and overall survival in men and women, estimated by period survival analysis, **D** trend of relative 5-year survival in men and women, estimated by period survival analysis

With a median follow-up of 8.8 years, the 5-year OS was 69.6% (95% CI 67.6–71.6%) in men and 80.4% (95% CI 80.3–80.6%) in women (*p* < 0.001, Fig. 2A). According to the univariable Cox regression, the risk of death was 1.76 times greater in men than in women (95% CI 1.65–1.87; *p* < 0.001). In the multivariable analysis, men still had a 1.32-fold increased risk of death (95% CI 1.24–1.41; *p* < 0.001) (Table 2). In addition to sex, the following variables significantly increased the risk of death with and without adjustment: older age at diagnosis, bilateral side localization, other malignant neoplasms, higher stage, higher grading, lymphatic and venous invasion, and high-risk Ki-67 status. According to univariable and multivariable Cox regression after diagnosis, right-sided localization, positive hormone receptor status, and positive HER2 status significantly reduce the risk of death.

**Table 2:** Results of the Cox regression on the overall survival of patients in stages I–IV

		Univariable cox regression				Multivariable cox regression			
Variable	Category	p^a^	HR	95% CI for HR		p	HR	95% CI for HR	
				Lower limit	Upper limit			Lower limit	Upper limit
Sex	Women	<.001	Ref.^b^			<.001	Ref		
	Men	<.001	1.76	1.65	1.87	.001	1.32	1.24	1.41
Period of diagnosis	2000–05	<.001	Ref			<.001	1.00		
	2006–11	<.001	.85	.83	.86	<.001	.94	.92	.96
	2012–18	<.001	.71	.70	.72	<.001	.77	.75	.80
Age at diagnosis (years)	0–49	<.001	Ref			<.001	Ref		
	50–59	<.001	1.09	1.06	1.12	<.001	1.17	1.14	1.21
	60–69	<.001	1.49	1.45	1.53	<.001	1.63	1.59	1.67
	70–79	<.001	3.16	3.09	3.24	<.001	3.09	3.02	3.17
	≥ 80	<.001	7.25	7.07	7.43	<.001	6.42	6.26	6.59
Side localization ICDO-3	Left	<.001	Ref			<.001	Ref		
	Right	<.001	.98	.96	.99	.047	.99	.97	1.00
	Both sides	<.001	4.53	3.47	5.92	<.001	1.64	1.25	2.14
	n.s.^c^	<.001	2.69	2.52	2.88	<.001	1.23	1.15	1.32
Histology classification	Invasive carcinoma	<.001	Ref			<.001	Ref		
	DCIS^d^	<.001	.66	.55	.81	.001	.70	.57	.86
	Other malignant neoplasms	<.001	1.97	1.86	2.08	.003	1.09	1.03	1.16
Stage UICC	I	<.001	Ref			<.001	Ref		
	0	.309	1.06	.95	1.19	.748	.98	.87	1.11
	II	<.001	2.03	1.99	2.08	<.001	1.65	1.62	1.69
	III	<.001	4.40	4.30	4.50	<.001	3.12	3.04	3.19
	IV	<.001	14.65	14.32	14.98	<.001	9.98	9.74	10.22
	X/n. s	<.001	4.78	4.66	4.91	<.001	2.78	2.70	2.86
Grading	G1	<.001	Ref			<.001	Ref		
	G2	<.001	1.67	1.62	1.71	<.001	1.18	1.15	1.22
	G3/4	<.001	2.30	2.24	2.37	<.001	1.39	1.35	1.43
	GX/n. s	<.001	3.24	3.13	3.36	<.001	1.41	1.36	1.47
Lymphatic invasion	0	<.001	Ref			<.001	Ref		
	1	<.001	2.35	2.31	2.40	<.001	1.23	1.20	1.25
	X/n. s	<.001	2.16	2.12	2.19	<.001	1.12	1.09	1.15
Venous invasion	0	<.001	Ref			<.001	Ref		
	1	<.001	2.84	2.75	2.92	<.001	1.26	1.22	1.30
	X/n. s	<.001	1.77	1.74	1.79	<.001	1.22	1.19	1.25
Hormone receptor (HR) status	Negative	<.001	Ref			<.001	Ref		
	Positive	<.001	.72	.70	.73	<.001	.69	.68	.71
	n. s	<.001	.93	.90	.95	<.001	.72	.70	.74
HER2 status	Negative	<.001	Ref			<.001	Ref		
	Positive	<.001	1.08	1.06	1.10	<.001	.93	.91	.95
	n. s	<.001	1.35	1.33	1.37	<.001	1.10	1.08	1.12
Ki-67	Low risk (< 25%)	<.001	Ref			<.001	Ref		
	high risk (≥ 25%)	<.001	1.71	1.66	1.77	<.001	1.33	1.29	1.38
	n. s	<.001	1.59	1.56	1.63	<.001	1.17	1.14	1.20

^a^The p-values in the table given in the reference category refer to the entire variable

^b^Ref.: Reference

^c^N. s.: not specified

^d^Carcinoma in situ diagnosed after neoadjuvant therapy

### Relative and period survival

Applying age, sex and calendar year specific German life tables from the years 2000 to 2018, 5-year RS in men was 83.0% (95% CI 80.6–85.4%) vs. 88.1% (95%-CI 87.9–88.2%) in women. The direct comparison of the OS and RS survival curves in Fig. 2B underlines the more pronounced difference in men between both endpoints, reflecting the lower life expectancy in men compared to women.

Period survival analysis yielded better estimates for both OS and RS, giving more weight to the current years (Fig. 2C): 5-year OS increased to 72.7% (95% CI 69.5–75.8%) in men, RS increased to 87.3% (95% CI 83.5–91.1%). The period analysis in women showed an increase to a 5-year OS of 83.0% (95% CI 82.8%- 83.3%) and a 5-year RS of 91.2% (95% CI 90.9–91.5). As to consecutive periods in Fig. 2D the constant improvement of relative survival is more distinctly in men compared to women, reducing the difference in survival between men and women in later years.

### Overall survival in subgroups

In the cohort without primary distant metastases (patients with stage I–III disease and residual tumor R0) (n = 1881 men, n = 240,737 women), a worse outcome for men was observed for all endpoints after risk adjustment (Table 3). The 5-year OS without primary distant metastases was 76.7% in men and 87.3% in women (*p* < 0.001).

**Table 3:** Overall survival and cumulative recurrence rate by sex (results of multivariable Cox regression adjusted for period of diagnosis, age, side localization, histology, stage, grading, lymphatic/venous invasion, hormone receptor status, HER2 status and Ki-67)

	Sex	5-year rate (%)	Chi Square p	Log-rank p^a^	Hazard ratio (HR)	95% CI for HR	
						Lower limit	Upper limit
Overall survival (stage I–IV)	Women	80.4			Ref.^b^		
	Men	69.6	< 0.001	<.001	1.32	1.24	1.41
Overall survival (stage I–III and R0)	Women	87.3			Ref		
	Men	76.7	< 0.001	< 0.001	1.48	1.37	1.60
Cumulative recurrence rate (stage I–III and R0)	Women	10.8			Ref		
	Men	17.9	< 0.001	< 0.001	1.49	1.34	1.66
Cumulative locoregional recurrence rate (stage I–III and R0)	Women	3.2			Ref		
	Men	4.0	0.113	0.022	1.30	1.04	1.63
Cumulative distant metastatic recurrence rate (stage I–III and R0)	Women	8.0			Ref		
	Men	14.0	< 0.001	< 0.001	1.52	1.35	1.71
Recurrence-free survival (stage I–III and R0)	Women	82.4			Ref		
	Men	67.6	< 0.001	< 0.001	1.53	1.43	1.65

^a^The p-values in the table given in the reference category refer to the entire variable

^b^Ref.: Reference

In prognostically favorable subgroups such as low-stage, low-grade, absent lymphatic invasion, positive hormone receptor status, and Ki-67 < 25%, survival tended to be worse for men than for women in the overall cohort without primary distant metastases. In earlier years of diagnosis (< 2005) and prognostically unfavorable subgroups such as older age at diagnosis, higher stage, G2 grade and lymphatic invasion, the difference in the risk of death between men and women tended to be smaller. In later years after diagnosis (≥ 2006), survival tended to be worse for men than for women (Table 4). Adjustment for therapy in a multivariable regression model did not significantly affect the risk of death between men and women (Supp. Table E). Notably, systemic therapies had a comparable positive effect on OS in both sexes (Supp. Table F).

**Table 4:** Results of the Cox regression for the overall survival of men versus women in subgroups according to patient and tumor characteristics (in patients with stage I–III, without primary distant metastases)

		Multivariable cox regression				HR in this subgroup lower ↓ (italic) or higher ↑ (bold) in comparison to HR in all patients with stage I-III and R0
Variable	Category	p^a^	HR	95% CI for HR		
				Lower limit	Upper limit	
Period of diagnosis	2000–05	<.001	*1.39*	1.23	1.57	↓
	2006–11	<.001	**1.53**	1.35	1.73	↑
	2012–18	<.001	**1.61**	1.34	1.92	↑
Age at diagnosis (years)	0–49	.002	**1.87**	1.26	2.77	↑
	50–59	.002	**1.51**	1.16	1.96	↑
	60–69	<.001	**1.70**	1.47	1.98	↑
	70–79	<.001	*1.40*	1.24	1.59	↓
	≥ 80	.003	*1.31*	1.10	1.57	↓
Side localization ICDO-3	Left	<.001	**1.53**	1.37	1.70	↑
	Right	<.001	*1.42*	1.26	1.60	↓
	Both sides^b^	–	**–**	–	–	↑
	n. s.^c^	.004	**6.43**	1.82	22.67	↑
Histology classification	Invasive Carcinoma	<.001	*1.48*	1.36	1.60	↓
	DCIS^d^	.156	**7.17**	.47	109.39	↑
	Other malignant neoplasms	.308	**1.62**	.64	4.07	↑
Stage UICC	I	<.001	**1.58**	1.32	1.90	↑
	II	<.001	**1.62**	1.44	1.82	↑
	III	<.001	*1.31*	1.15	1.49	↓
Grading	G1	<.001	**1.65**	1.27	2.14	↑
	G2	<.001	*1.44*	1.29	1.60	↓
	G3/4	<.001	**1.51**	1.31	1.74	↑
	GX/n. s	.064	**1.50**	.98	2.30	↑
Lymphatic invasion	0	<.001	**1.59**	1.39	1.82	↑
	1	<.001	*1.35*	1.19	1.53	↓
	X/n. s	<.001	**1.60**	1.37	1.86	↑
Venous invasion	0	<.001	*1.46*	1.32	1.62	↓
	1	.004	**1.58**	1.16	2.15	↑
	X/n. s	<.001	**1.50**	1.31	1.71	↑
Hormone receptor (HR) status	Negative	<.001	**2.08**	1.42	3.03	↑
	Positive	<.001	**1.48**	1.36	1.62	↑
	n. s	<.001	*1.45*	1.21	1.75	↓
HER2 status	Positive	<.001	**2.16**	1.70	2.74	↑
	Negative	<.001	*1.42*	1.29	1.57	↓
	n. s	<.001	*1.43*	1.21	1.70	↓
Ki-67	Low risk (< 25)	<.001	**1.76**	1.39	2.23	↑
	High risk (≥ 25)	.105	*1.27*	.95	1.71	↓
	n. s	<.001	*1.48*	1.35	1.61	↓

^a^The p-values in the table given in the reference category refer to the entire variable

^b^No identification of men with bilateral breast cancer

^c^N. s.: not specified

^d^Carcinoma in situ diagnosed after neoadjuvant therapy

### Recurrence rate and RFS in stage I–III and R0 patients

The 5-year cumulative overall recurrence rate was 17.9% in men and 10.8% in women (Table 3). The log-rank test revealed no significant difference in the rate of locoregional recurrence between men and women (5-year cumulative rate: m: 4.0%, w: 3.2%; *p* = 0,113) but did reveal a significant difference in the rate of distant metastasis recurrence (5-year cumulative rate: m: 14.0%, w: 8.0%; *p* < 0,001).

There was a significant difference in RFS between men and women (5-year cumulative RFS rate: m:67.6%, w: 82.4%; *p* < 0.001). According to the univariable Cox regression, the risk of death or recurrence was twofold greater in men than in women. After multivariable adjustment, the risk of recurrence/mortality was lower but still statistically significant, with an HR of 1.53 (95% CI 1.43- 1.65; *p* < 0.001) for men compared with women.

### Rate of contralateral second tumors in stage I-IV patients

Among the 295,594 patients with ICD-10 C50, 1.0% (n = 24) of the men and 2.3% (n = 6641) of the women had simultaneous contralateral second tumors, and 0.4% (n = 9) of the men and 1.9% (n = 5606) of the women had subsequent contralateral second tumors (Supp. Table G). According to univariable and multivariable logistic regression, men had a significantly lower risk of simultaneous (multivariable: OR = 0.33, 95% CI 0.22–0.49; *p* < 0.001) and subsequent (multivariable: HR = 0.24, 95% CI 0.125–0.464; *p* < 0.001) contralateral second tumors than women did. Multivariable Cox regression revealed similar results for subsequent contralateral secondary tumors (HR = 0.27, 95% CI 0.14–0.52; *p* < 0.001) (Supp. Table H).

## Discussion

In the present study, there were significant differences between male and female breast cancer patients in terms of patient and tumor characteristics. In summary, men presented a greater risk for unfavorable prognostic parameters, which can be attributed to several factors. As breast cancer is rare in men, there might be a lack of awareness and knowledge about it. Moreover, there is no screening for the early detection of breast cancer in men (Aid and AWMF 2021). Male patients had a greater proportion of luminal B tumors, whereas female patients had higher proportions of luminal A, HER2-positive and triple-negative tumors. These findings agree with a retrospective study using data from the pathology archives of Memorial Sloan Kettering Cancer Center (n = 59), where men primarily had luminal B tumors (Piscuoglio et al. 2016), whereas other studies reported that the most common subtype in women was luminal A (Inwald et al. 2017, 2015). Similar to our study, numerous studies have revealed that positive estrogen receptor status is more prevalent in men (Giordano et al. 2004; Anderson et al. 2010; Elimimian et al. 2021; Gnerlich et al. 2011; Aggarwal et al. 2021; Liu et al. 2018; Wang et al. 2019). Various studies have shown that male breast cancer patients are more often older at diagnosis (Miao et al. 2011; Giordano et al. 2004; Anderson et al. 2010; Elimimian et al. 2021; Gnerlich et al. 2011; Aggarwal et al. 2021; Liu et al. 2018; Wang et al. 2019) and have a higher stage (Giordano et al. 2004; Elimimian et al. 2021; Gnerlich et al. 2011; Aggarwal et al. 2021; Joshi et al. 1996; Liu et al. 2018; Wang et al. 2019) and lymphatic invasion (Gnerlich et al. 2011; Brenner and Hakulinen 2002) than women, which is consistent with our results. A registry-based cohort study by Wang et al. utilizing data from the National Cancer Database (NCDB) between 2004 and 2014 (n(male) = 16,025, n(female) = 1,800,708) revealed a mean age at diagnosis of 63.3 years in men and 59.9 years in women (Wang et al. 2019). There are no consistent results regarding grading. A retrospective study by Elimimian et al. (data from NCDB; 2004–2016; n(men) = 23,990, n(women) = 23,990) reported higher grading in men than in women (Elimimian et al. 2021), whereas a retrospective cohort study with data from the Surveillance, Epidemiology, and End Results (SEER) program (1988–2003; n(men) = 1541, n(women) = 244,518) reported the opposite (Gnerlich et al. 2011). Our study revealed no significant differences. The occurrence of primary distant metastases was found to be more common among men. This finding was corroborated by a retrospective study by Giordano et al. analyzing the SEER data of 2537 men and 383,146 women between 1973 and 1998 (Giordano et al. 2004). The increased risk of distant metastases in the lung in men compared to women could possibly be explained by the risk factor smoking, which is associated with an increased risk of lung metastases in women with breast cancer (Scanlon et al. 1995).

In our study, the five-year OS was significantly lower for men than for women (69.6% vs. 80.4%, p < 0.001). The unadjusted risk of death in male patients was 1.76 times higher than that in female patients, which is in accordance with data from the RKI (Ronckers et al. 2023) and findings of studies by Wang and Elimimian, who reported a shorter survival rate for men with breast cancer (Elimimian et al. 2021; Wang et al. 2019). In a further population-based study, the HR was 1.75 (males vs. females) without adjustment (Elimimian et al. 2021). In a study by Gnerlich et al., the overall mortality rate of men in each stage was higher than that of women. The breast cancer-specific risk of death in stages II–IV did not differ between the sexes, but in stage I, there was a higher breast cancer-specific risk of death in men than in women. Giordano et al. reported that male 5-year survival rates at each stage were lower than those of females. However, the relative survival rates of men and women did not differ when the life expectancy of the population was considered, depending on race, sex and age (Giordano et al. 2004). In contrast, the present study showed that male patients had lower overall and relative survival than female patients. When relative survival was calculated, the survival difference between men and women was smaller compared to the overall survival difference, but there was still a remaining difference. The difference tended to be smaller in more recent 5-year relative survival periods (Fig. 2D). The results from Suppl. Table A show an increase in male breast cancer diagnoses from 2012 to 2018 compared to 2000–2005. This could be due to a higher awareness and lead to earlier diagnosis and earlier treatment reducing the difference in survival between men and women in more recent years. In a retrospective study by Miao et al. with data from population-based cancer registries in Denmark, Finland, Geneva, Norway, Singapore, and Sweden (n(men) = 2665, n(women) = 459,846), the OS of men without distant metastases and the breast cancer-specific survival of men were worse than those of women. After adjustment for region, age, period of diagnosis, follow-up time, stage, and therapy, men had better breast cancer-specific survival than women did (Liu et al. 2018). In our study, even after adjusting for age at diagnosis, side of localization, histology, stage, grading, lymphatic/venous invasion, hormone receptor status, HER2 status, Ki-67, and period of diagnosis, the risk of death in males was 1.32 times greater than that in females (p < 0.001). Similar results were shown in a study by Elimimian et al. After adjusting for age, stage, and comorbidity score, males were 1.25 times more likely to die. After further adjustment for race, income, insurance type, grade, ER and PR status, and tumor site, the risk of death in men remained 1.28 times greater than that in women (Elimimian et al. 2021). A further retrospective study including SEER data from 289,673 patients between 2005 and 2010 confirmed that the risk of death remained greater in men (HR 1.43) even after considering the age of diagnosis, race, marital status, stage, lymph node status, ER status, and geographic region (Liu et al. 2018). A study by Wang et al. revealed that OS was lower for males even after adjustment for multiple factors (stage, hormone receptor status, HER2 status, age, period of diagnosis, comorbidities, histological grading, lymphatic invasion, treatment, race/ethnicity, and access to medical care). Wang suspected that other prognostic factors could play a role (Wang et al. 2019). Even in the cohort without primary distant metastases (stages I–III and R0), men had poorer survival and recurrence rates (5-year survival rates of 76.7% in men and 87.3% in women), which is consistent with the findings of a study by Miao et al. (Miao et al. 2011). In our study, the 5-year cumulative rate of locoregional recurrence did not differ significantly between men and women. The rare occurrence of locoregional distant metastases in men could be explained by the higher rate of mastectomy in men. Compared with female patients, the risk for simultaneous or subsequent contralateral secondary tumors was significantly lower in male patients, even after multivariable adjustment. However the small numbers of cases and events in men leads to a low power of the risk estimation.

A retrospective study with data from the SEER program (1973–2005, n(male) = 5494, n(female) = 835,805)) revealed an improvement in survival over time, especially for women. This could be attributed to the fact that men may be treated later than women and may be less likely to receive the appropriate therapy (Anderson et al. 2010). Aggarwal et al. reported that men were more likely to receive endocrine therapy than women, whereas women were more likely to receive CHT or radiotherapy (Aggarwal et al. 2021). We examined systemic therapies in the respective treatment groups. For each indication group, the treatment rate for men was 9.5–29.0% lower than that for women. Similarly, Wang reported that endocrine therapy was performed less frequently in hormone receptor-positive men than in hormone receptor-positive women. He further examined radiotherapy in patients who underwent BCT or stage II and III patients who underwent mastectomy; even in this group, men received less radiation than women did. After adjustment for therapy, the risk of death in men remained higher than that in women (Wang et al. 2019). This is consistent with the results of our study. The consideration of the respective systemic therapy in the multivariable regression could not eliminate the difference in the risk of death between men and women. Furthermore, we found that men had a comparable survival benefit from systemic therapies to women.

## Limitations

The weaknesses of our study are especially the lack of consideration of specific factors that could explain the difference in survival between men and women, such as the exact tumor location with specification of the quadrant and the tumor size, which, in stage I, could differ between men and women. In addition, breast cancer-specific survival was not calculated and thus, differences in lifestyle (e.g., nicotine consumption, alcohol abuse) and life expectancy were neglected. Instead, however, relative survival rates were estimated. Further information on certain genes or other biological factors was also missing from our dataset.

Nevertheless, numerous patient and tumor characteristics were included in our study, and the different locations of primary distant metastases were also taken into account. Moreover, different therapies were considered for better comparability between men and women in the respective indication groups. There are few data regarding this topic in the literature. To our knowledge, the present study is the most comprehensive analysis of male breast cancer patients in Germany and one of the largest studies worldwide.

## Conclusion

Neither patient and tumor characteristics nor differences in therapy could completely explain the difference in the risk of death between men and women. Differences in lifestyle such as abuse of nicotine and alcohol, dietary habits, physical exercise and health consciousness or biological factors could play a role. Further studies should consider additional factors to determine the causes of the poorer prognosis in men and to find opportunities for improving the treatment of men with breast cancer.

## Supplementary Information

Below is the link to the electronic supplementary material.

Supplementary file1 (DOCX 82 KB)

## Data Availability

No datasets were generated during the current study. Data of the current study are not publicly available due to the data security of the cancer registries.

## Abbreviations

ADT: Association of german tumor centers (Arbeitsgemeinschaft Deutscher Tumorzentren)
BCT: Breast-conserving therapy
CHT: Chemotherapy
CI: Confidence interval
HER2: Human epidermal growth factor receptor 2
HR: Hazard ratio
ICD: International statistical classification of diseases and related health problems
NCDB: National cancer database
OR: Odds ratio
OS: Overall survival rate
RFS: Recurrence-free survival rate
RKI: Robert Koch institute
RS: Relative survival
SEER: Surveillance, epidemiology, and end results
UICC: Union internationale contre le cancer
