# Supplementary material for: Clinical, therapeutic and prognostic differences between male and female patients with breast cancer—a comparison of 2510 men and 307,634 women in a registry-based study in Germany
Source: J Cancer Res Clin Oncol. 2025 Jun 3;151(6):181. doi: 10.1007/s00432-025-06220-y (PMC12134001; doi:10.1007/s00432-025-06220-y)
Supplement: Supplementary file 1 — Supplementary file1 (DOCX 82 KB) [file 432_2025_6220_MOESM1_ESM.docx]

*Table A Distribution of patient and tumor characteristics by sex*

|  | | *Men* | | *Women* | | *Total* | | *p* |
| --- | --- | --- | --- | --- | --- | --- | --- | --- |
|  |  | *Number* | *%* | *Number* | *%* | *Number* | *%* |  |
| *Period of diagnosis* | *2000-05* | 608 | 24.2 | 83963 | 27.3 | 84571 | 27.3 | <.001 |
|  | *2006-11* | 771 | 30.7 | 104845 | 34.1 | 105616 | 34.1 |  |
|  | *2012-18* | 1131 | 45.1 | 118826 | 38.6 | 119957 | 38.7 |  |
| *Age at diagnosis (years)* | *0-49* | 186 | 7.4 | 58300 | 19.0 | 58486 | 18.9 | <.001 |
|  | *50-59* | 351 | 14.0 | 68263 | 22.2 | 68614 | 22.1 |  |
|  | *60-69* | 764 | 30.4 | 81467 | 26.5 | 82231 | 26.5 |  |
|  | *70-79* | 839 | 33.4 | 64779 | 21.1 | 65618 | 21.2 |  |
|  | *≥80* | 370 | 14.7 | 34825 | 11.3 | 35195 | 11.3 |  |
| *Side localization ICDO-3* | *Left* | 1346 | 53.6 | 157780 | 51.3 | 159126 | 51.3 | .030 |
|  | *Right* | 1146 | 45.7 | 148241 | 48.2 | 149387 | 48.2 |  |
|  | *both sides* | 0 | 0.0 | 79 | 0.0 | 79 | 0.0 |  |
|  | *n. s.^a^* | 18 | 0.7 | 1534 | 0.5 | 1552 | 0.5 |  |
| *Histology classification* | *Invasive carcinoma* | 2480 | 98.8 | 304666 | 99.0 | 307146 | 99.0 | .180 |
|  | *DCIS^b^* | 2 | 0.1 | 449 | 0.1 | 451 | 0.1 |  |
|  | *Other malignant neoplasms* | 28 | 1.1 | 2519 | 0.8 | 2547 | 0.8 |  |
| *Stage UICC* | *0* | 5 | 0.2 | 3117 | 1.0 | 3122 | 1.0 | <.001 |
|  | *I* | 581 | 23.1 | 116205 | 37.8 | 116786 | 37.7 |  |
|  | *II* | 905 | 36.1 | 107892 | 35.1 | 108797 | 35.1 |  |
|  | *III* | 549 | 21.9 | 39036 | 12.7 | 39585 | 12.8 |  |
|  | *IV* | 248 | 9.9 | 21495 | 7.0 | 21743 | 7.0 |  |
|  | *X/ n. s.* | 222 | 8.8 | 19889 | 6.5 | 20111 | 6.5 |  |
| *Grading* | *G1* | 277 | 11.0 | 42382 | 13.8 | 42659 | 13.8 | <.001 |
|  | *G2* | 1449 | 57.7 | 166132 | 54.0 | 167581 | 54.0 |  |
|  | *G3* | 641 | 25.5 | 85969 | 27.9 | 86610 | 27.9 |  |
|  | *GX/ n. s.* | 143 | 5.7 | 13151 | 4.3 | 13294 | 4.3 |  |
| *Lymphatic invasion* | *0* | 997 | 39.7 | 150113 | 48.8 | 151110 | 48.7 | <.001 |
|  | *1* | 811 | 32.3 | 68045 | 22.1 | 68856 | 22.2 |  |
|  | *X/ n. s.* | 702 | 28.0 | 89476 | 29.1 | 90178 | 29.1 |  |
| *Venous invasion* | *0* | 1536 | 61.2 | 192433 | 62.6 | 193969 | 62.5 | <.001 |
|  | *1* | 126 | 5.0 | 10007 | 3.3 | 10133 | 3.3 |  |
|  | *X/ n. s.* | 848 | 33.8 | 105194 | 34.2 | 106042 | 34.2 |  |
| *Hormon receptor (HR) status* | *Positive* | 1966 | 78.3 | 214114 | 69.6 | 216080 | 69.7 | <.001 |
|  | *Negative* | 71 | 2.8 | 39190 | 12.7 | 39261 | 12.7 |  |
|  | *n. s.* | 473 | 18.8 | 54330 | 17.7 | 54803 | 17.7 |  |
| *HER2 status* | *Positive* | 212 | 8.4 | 36635 | 11.9 | 36847 | 11.9 | <.001 |
|  | *Negative* | 1839 | 73.3 | 220292 | 71.6 | 222131 | 71.6 |  |
|  | *n. s.* | 459 | 18.3 | 50707 | 16.5 | 51166 | 16.5 |  |
| *Ki-67* | *Low risk (<25%)* | 582 | 23.2 | 69666 | 22.6 | 70248 | 22.7 | .219 |
|  | *High risk (≥25%)* | 347 | 13.8 | 39539 | 12.9 | 39886 | 12.9 |  |
|  | *n.s.* | 1581 | 63.0 | 198429 | 64.5 | 200010 | 64.5 |  |
| *Molecular subtypes* | *Luminal A* | 304 | 12.1 | 43098 | 14.0 | 43402 | 14.0 | <.001 |
|  | *Luminal B* | 435 | 17.3 | 30153 | 9.8 | 30588 | 9.9 |  |
|  | *HER2 enriched* | 212 | 8.4 | 36635 | 11.9 | 36847 | 11.9 |  |
|  | *Triple negative* | 45 | 1.8 | 25590 | 8.3 | 25635 | 8.3 |  |
|  | *n. s.* | 1514 | 60.3 | 172158 | 56.0 | 173672 | 56.0 |  |
| *Total* | | 2510 | 100.0 | 307634 | 100.0 | 310144 | 100.0 |  |

*^a^ N.s.: not specified*

*b Carcinoma in situ diagnosed after neoadjuvant therapyTable B Sentinel lymph node biopsy in patients with clinically inconspicious lymph nodes (cN0)*

| *Sentinel lymph node biopsy* | *male patients* | | *female patients* | | *total* | |
| --- | --- | --- | --- | --- | --- | --- |
|  | *number* | *%* | *number* | *%* | *Number* | *%* |
| *Performed* | 309 | 88.3 | 60884 | 89.8 | 61193 | 89.8 |
| *not performed* | 41 | 11.7 | 6936 | 10.2 | 6977 | 10.2 |
| *Total* | 350 | 100.0 | 67820 | 100.0 | 68170 | 100.0 |

Table C HER2 analysis in male and female patients

| *HER2 examination* | *male patients* | | *female patients* | | *total* | |
| --- | --- | --- | --- | --- | --- | --- |
|  | *number* | *%* | *number* | *%* | *number* | *%* |
| *Performed* | 2051 | 81.7 | 256927 | 83.5 | 258978 | 83.5 |
| *not performed* | 459 | 18.3 | 50707 | 16.5 | 51166 | 16.5 |
| *Total* | 2510 | 100.0 | 307634 | 100.0 | 310144 | 100.0 |

Table D Surgery performed in male and female patients

| *Surgery* | *Male patients* | | *Female patients* | | *Total* | |
| --- | --- | --- | --- | --- | --- | --- |
|  | *number* | *%* | *number* | *%* | *Number* | *%* |
| *BCT* | 261 | 13.9 | 173623 | 72.1 | 173884 | 71.7 |
| *Mastectomy* | 1566 | 83.3 | 59712 | 24.8 | 61278 | 25.3 |
| *Other surgery/ not specified* | 51 | 2.7 | 6030 | 2.5 | 6081 | 2.5 |
| *No surgery* | 3 | 0.2 | 1372 | 0.6 | 1375 | 0.6 |
| *Total* | 1881 | 100.0 | 240737 | 100.0 | 242618 | 100.0 |

Table E Multivariable Cox regression with and without adjustment for therapy in patients with respective indication. The collective was restricted to patients in stages I – III with local R0 status after BET or mastectomy. Indication for endocrine therapy was a positive hormone receptor status. Analyses for antibody therapy were only carried out from 2006, since the proportion of testing for HER2 status in previous years was very low. Indication for antibody therapy and antibody and chemotherapy was a positive HER2 status. Indication for chemotherapy was a negative hormone receptor status and/or positive lymph nodes and/or grading G3/4 and/or under 35 years of age.

|  |  |  | *Without adjustment for therapy* | | | | *With adjustment for therapy* | | | |
| --- | --- | --- | --- | --- | --- | --- | --- | --- | --- | --- |
| *Therapy* | *Sex* | *n (patients with indication)* | *p^a^* | *HR* | *95%-CI* | | *p* | *HR* | *95%-CI* | |
|  |  |  |  |  | *Lower limit* | *Upper limit* |  |  | *Lower limit* | *Upper limit* |
| *Endocrine therapy* | *women* | *173,008* |  | Ref^b^ |  |  |  | Ref. |  |  |
|  | *men* | *1,543* | < .001 | 1.49 | 1.36 | 1.63 | < .001 | 1.47 | 1.34 | 1.61 |
| *Antibody therapy* | *women* | *2,2012* |  | Ref. |  |  |  | Ref. |  |  |
|  | *men* | *133* | < .001 | 2.45 | 1.85 | 3.25 | < .001 | 2.37 | 1.79 | 3.14 |
| *Antibody and chemotherapy* | *women* | *2,2012* |  | Ref. |  |  |  | Ref. |  |  |
|  | *men* | *133* | < .001 | 2.45 | 1.85 | 3.25 | < .001 | 2.33 | 1.76 | 3.09 |
| *Chemo-therapy* | *women* | *120,269* |  | Ref. |  |  |  | Ref. |  |  |
|  | *men* | *1,001* | < .001 | 1.48 | 1.34 | 1.64 | < .001 | 1.49 | 1.35 | 1.65 |

*The p-values in the tables given in the reference category refer to the entire variable.*

*^b^ Ref.: Reference*

Table F Multivariable Cox regression for overall survival in dependence of therapy applied yes vs no separated by sex in patients with respective indication

| *Therapy* | *Sex* | *p^a^* | *HR* | *95%-CI* | |
| --- | --- | --- | --- | --- | --- |
|  |  |  |  | *Lower limit* | *Upper limit* |
| *Endocrine therapy* | *Women* | < .001 | .75 | .73 | .77 |
|  | *Men* | < .001 | .71 | .58 | .86 |
| *Antibody therapy* | *Women* | < .001 | .63 | .58 | .68 |
|  | *Men* | .245 | .63 | .29 | 1.37 |
| *Chemotherapy* | *Women* | < .001 | .75 | .73 | .77 |
|  | *Men* | .002 | .70 | .56 | .88 |

^a^ The p-values in the tables given in the reference category refer to the entire variable.

Table G Rate of contralateral secondary tumors in the group of 2,467 male patients and 293,127 female patients

|  | *Male patients* | | *Female patients* | | *Total* | |
| --- | --- | --- | --- | --- | --- | --- |
|  | *number* | *%* | *number* | *%* | *number* | *%* |
| *Simultanous contralateral secondary tumors* | 24 | 1.0 | 6641 | 2.3 | 6665 | 2.3% |
| *Subsequent contralateral secondary tumors* | 9 | 0.4 | 5606 | 1.9 | 5615 | 1.9% |

*Table H Uni- and multivariable logistic regression with OR and multivariable Cox regression with HR men versus women for simultanous and subsequent contralateral secondary tumors*

|  | *Sex* | *Log-rank p^a^* | *Odds Ratio (OR) or Hazard ratio (HR)* | *95% CI für HR* | |
| --- | --- | --- | --- | --- | --- |
|  |  |  |  | *Lower limit* | *Upper limit* |
| *Univariable logistic regression* | | | | | |
| *Simultanous contralateral secondary tumors* | *Women* | < .001 | Ref.^b^ |  |  |
|  | *Men* | < .001 | .42 | .28 | .63 |
| *Subsequent contralateral secondary tumors* | *Women* | < .001 | Ref. |  |  |
|  | *Men* | < .001 | .19 | .10 | .36 |
| *Multivariable logistic regression* | | | | | |
| *Simultanous contralateral secondary tumors* | *Women* | < .001 | Ref. |  |  |
|  | *Men* | < .001 | .33 | .22 | .49 |
| *Subsequent contralateral secondary tumors* | *Women* | < .001 | Ref. |  |  |
|  | *Men* | < .001 | .24 | .13 | .46 |
| *Multivariable Cox regression* | | | | | |
| *Simultanous contralateral secondary tumors* | *Women* | **-** | **-** | **-** | **-** |
|  | *Men* | **-** | **-** | **-** | **-** |
| *Subsequent contralateral secondary tumors* | *Women* | < .001 | Ref. |  |  |
|  | *Men* | .000 | .27 | .14 | .52 |

*^a^ The p-values in the tables given in the reference category refer to the entire variable.*

*^b^ Ref.: Reference*


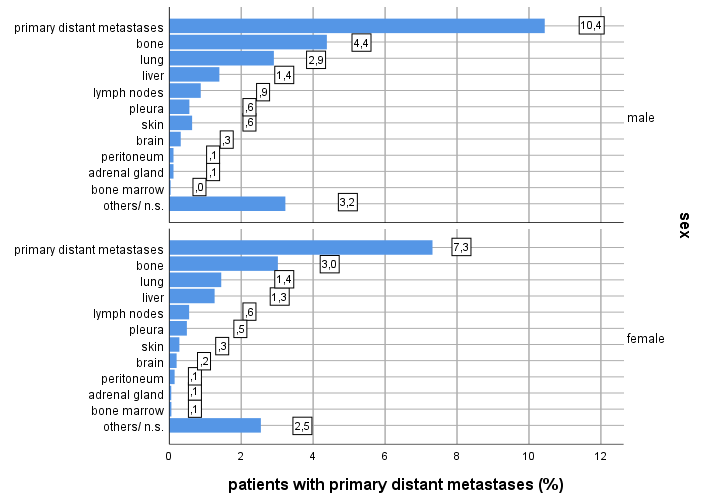


Figure A Primary distant metastases (%) of 2510 male and 307634 female patients (total and by location)


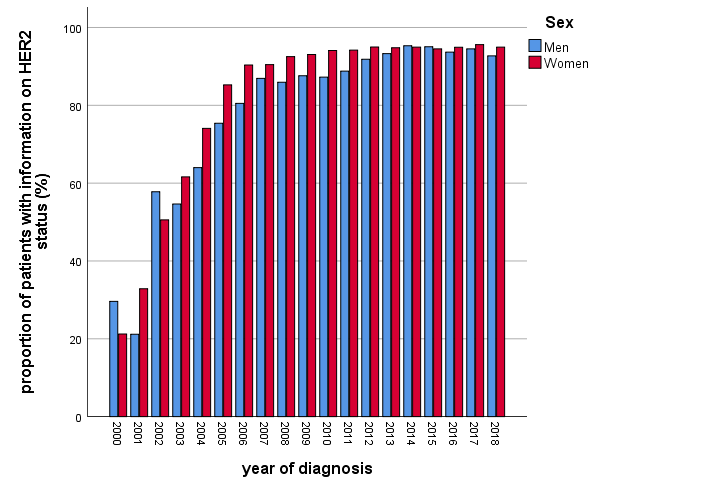


Figure B Proportion of men and women with HER2 testing by year of diagnosis
